# Tool Wear Prediction in Ti-6Al-4V Machining through Multiple Sensor Monitoring and PCA Features Pattern Recognition

**DOI:** 10.3390/s18030823

**Published:** 2018-03-09

**Authors:** Alessandra Caggiano

**Affiliations:** 1 Fraunhofer Joint Laboratory of Excellence on Advanced Production Technology (Fh-J_LEAPT UniNaples), P.le Tecchio 80, 80125 Naples, Italy; alessandra.caggiano@unina.it; Tel.: +39-081-7682371; Fax: +39-081-7682362; 2 Department of Industrial Engineering, University of Naples Federico II, P.le Tecchio 80, 80125 Naples, Italy

**Keywords:** turning, titanium alloy, tool condition monitoring, sensor fusion, dimensionality reduction, principal component analysis, machine learning, artificial neural network

## Abstract

Machining of titanium alloys is characterised by extremely rapid tool wear due to the high cutting temperature and the strong adhesion at the tool-chip and tool-workpiece interface, caused by the low thermal conductivity and high chemical reactivity of Ti alloys. With the aim to monitor the tool conditions during dry turning of Ti-6Al-4V alloy, a machine learning procedure based on the acquisition and processing of cutting force, acoustic emission and vibration sensor signals during turning is implemented. A number of sensorial features are extracted from the acquired sensor signals in order to feed machine learning paradigms based on artificial neural networks. To reduce the large dimensionality of the sensorial features, an advanced feature extraction methodology based on Principal Component Analysis (PCA) is proposed. PCA allowed to identify a smaller number of features (*k* = 2 features), the principal component scores, obtained through linear projection of the original *d* features into a new space with reduced dimensionality *k* = 2, sufficient to describe the variance of the data. By feeding artificial neural networks with the PCA features, an accurate diagnosis of tool flank wear (*VB_max_*) was achieved, with predicted values very close to the measured tool wear values.

## 1. Introduction

Titanium alloys offer exceptional properties, such as high strength-to-weight ratio, intermediate density, unique resistance to corrosion, low coefficient of thermal expansion and high toughness, which make these alloys extremely interesting for advanced applications in different sectors such as the aerospace, automotive and medical industries [1,2].

In the aerospace sector, the property of maintaining high strength at high operating temperatures makes Ti alloys suitable materials for aircraft engine components as well as for airframe structures (where these alloys can stand temperatures >130 °C, over the maximum for aluminium alloys) [2]. Moreover, Ti alloys are characterized by high compatibility with carbon fibre reinforced composite materials and are therefore increasingly employed in modern aircrafts [1].

However, the machinability of Ti alloys is generally poor due to the inherent material properties. Machining is characterised by extremely rapid tool wear and short tool life due to the high cutting temperature and the strong adhesion at the tool-chip and tool-workpiece interface, caused by the low thermal conductivity and high chemical reactivity of Ti alloys [2].

Moreover, their high strength preserved at elevated temperature and the low modulus of elasticity, responsible for workpiece bending, further impair Ti alloys machinability [3,4]. 

As regards the cutting speed, affecting temperature and tool wear, Ti alloys are generally difficult to machine under cutting speeds >30 m/min with high speed steel (HSS) tools and under cutting speeds >60 m/min with cemented tungsten carbide (WC) tools [4].

Due to the low-machinability of titanium alloys, the use of sensor systems for on-line monitoring of machining processes can significantly enhance the process performance in the perspective of zero defect manufacturing, by increasing productivity and reducing production costs [5,6,7,8]. 

Given the rapid development of tool wear and the unpredictable occurrence of catastrophic tool failure in Ti-machining, the implementation of on-line tool condition monitoring procedures may allow to optimise tool life by implementing smart strategies such as condition-based tool replacement instead of conservative time-based tool replacement [5,9,10,11].

In the literature, a number of research works have been developed with the aim to monitor the tool conditions based on relevant sensor signals acquired during the machining process. Kosaraju et al. [12] presented a procedure based on the acquisition of acoustic emission (AE) signals for tool wear prediction in the turning of titanium alloy. In Reference [13], Jemielniak discussed the main challenges related to the employment of acoustic emission sensors in tool condition monitoring, which requires an effective pre-processing of the high-frequency signals in order to reduce background noise, e.g., through the use of appropriate filters in the pre-amplifiers. Often, the raw AE signal is demodulated in the form of root mean square to obtain a low frequency variable that can be more conveniently processed. Jie et al. [14] investigated the effectiveness of cutting force and acoustic emission sensor signals, widely employed for machining process monitoring, with the aim to develop a tool condition monitoring system for turning of titanium alloy. Stavropoulos et al. [15] presented a methodology based on the simultaneous detection of acceleration and spindle drive current sensor signals for tool wear prediction in milling of CGI 450 plates, by utilizing third degree regression models and pattern recognition systems. Jemelniak et al. [16,17] proposed methodologies based on the acquisition and processing of acoustic emission sensor signals for the detection of catastrophic tool failures, i.e., sudden tool breakage events. With the same scope, Balsamo et al. [18] proposed a new methodology based on the acquisition of multiple sensor signals of different nature, including force as well as acoustic emission sensor signals. In [11,19], a methodology for a comprehensive tool condition monitoring, based on the acquisition and processing of multiple sensor signals to acquire a number of sensor signal features relevant for the monitoring of tool wear level and tool breakage events was presented. When using multiple sensors, the number of sensorial features extracted from the acquired signals can be high and a suitable selection method must be applied to reduce their dimensionality and lower the problem complexity. One of the most established methods for feature dimensionality reduction is principal components analysis, which is highly effective for the purpose of eliminating redundant and irrelevant information. This method was employed in [20] to reduce the dimensionality of cutting force and acoustic emission sensorial data for tool wear prediction in machining of mild steel. 

In this work, with the aim to monitor the tool conditions during dry turning of Ti-6Al-4V alloy, which is particularly challenging due to the poor machinability of the alloy, compared to steel and the absence of cutting fluid, an advanced sensor monitoring procedure via principal component analysis and machine learning is proposed. The procedure is based on the acquisition and processing of cutting force, acoustic emission and vibration sensor signals during turning by means of a multiple sensor system. A number of sensorial features are extracted from the acquired sensor signals in order to feed machine learning paradigms based on artificial neural networks for tool wear diagnosis. To reduce the large dimensionality of the sensorial features, which negatively affects the complexity and robustness of the machine learning algorithm, an advanced feature extraction methodology based on principal component analysis is proposed. The dimensionality reduction performed on the sensorial features data set allows to improve the efficiency of the machine learning procedure, by lowering the complexity of the models and drastically reducing the number of data to be stored and managed, which is a major goal taking into account the “big data” issues characterising today’s manufacturing in the Industry 4.0 framework, where a large amount of digital and sensorial data coming from the shop floor are continuously collected and processed.

## 2. Materials and Methods

### 2.1. Experimental Setup

To construct the training set for the machine learning procedure, experimental turning tests under diverse cutting conditions were carried out on grade 5 Ti-6Al-4V alloy bars with diameter of 60 mm. Each test was performed under a given cutting condition and consisted of repeated cylindrical turning passes, all having a length of 100 mm, executed with the same cutting tool until reaching complete tool wear. The total number of passes was different for each test, depending on the cutting conditions, as shown in Table 1.

The selected cutting tool was an uncoated tungsten carbide turning insert (Mitsubishi CNMG120404-MS MT9015) with ISO S15 carbide grade composition, suitable for titanium machining at intermediate material removal conditions. With the aim to investigate dry turning, no coolant was employed during the turning tests.

The experimental turning campaign was planned by varying the cutting parameters as follows: one level of cutting speed (*v* = 60 m/min), three levels of feed rate (*f* = 0.20, 0.25, 0.30 mm/rev) and three levels of depth of cut (*d* = 0.5, 1.0, 1.5 mm), as shown in Table 1.

**Figure 1:**
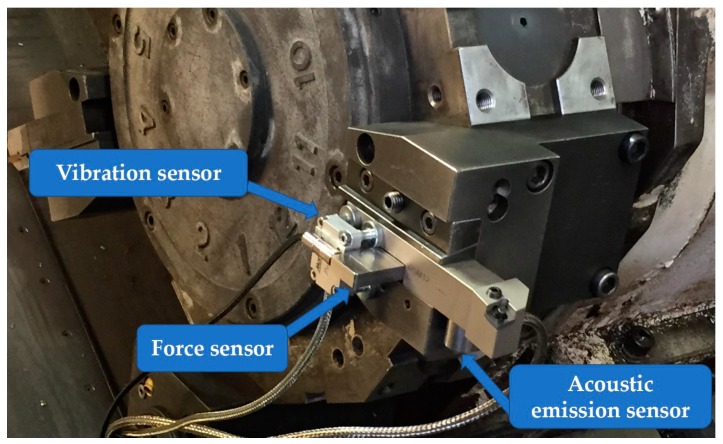
Sensor monitoring system mounted on the tool holder.

The turning tests were performed on a Daewoo Puma 400 LM CNC machine tool equipped with a multiple sensor system mounted on the tool holder to acquire relevant sensor signals during the process close to the tool-workpiece contact zone. The multi-sensor system, shown in Figure 1, consisted of a triaxial force sensor (Montronix FS1xCXK-x-ICA), an acoustic emission sensor (Montronix BV100) and a wireless digital triaxial vibration acceleration sensor (Montronix Spectra Pulse). As regards the acoustic emission signal, in order to obtain a low frequency variable, the AE sensor was set to acquire the root mean square (RMS) signal (*AE_RMS_*). As a matter of fact, raw acoustic emission signals, for which the frequency range could reach 1 MHz, require a high sampling frequency (>1 MS/s) and filtering of the analogue signal, both to respect the range of sensor frequency response and avoid high frequency noise, might be challenging. The broadband acoustic emission sensor was connected to a Montronix TSVA4G-BV amplifier embedding a RMS converter, a gain selection unit and filters. During the experimental tests, the amplifier was set up to acquire the acoustic emission RMS signals with a conversion time constant of 0.12 ms. The gain of the *AE_RMS_* signals was set to 2 in order to properly visualize the signals without exceeding the maximum threshold of 10 V imposed by the data acquisition (DAQ) board. The output low-pass filter cut-off frequency was set to high and a filter network module of 30 kHz–500 kHz was employed in the amplifier socket. The triaxial force sensor was connected to a Montronix TSFA3-ICA amplifier having 3-channel inputs, with gain settings equal to 10 for each channel and sensor range set to low gain, providing as output three raw signals, one for each force component. The signals coming from the first two analogue sensors were amplified and digitized through a NI USB-6361 DAQ board at a sampling rate of 10 kS/s and then sent to a PC. The employed DAQ board was a National Instruments NI USB-6361 device with an analogue to digital conversion (AC) resolution of 16 bits and a multichannel maximum (aggregate) sample rate of 1.00 MS/s. The output of the DAQ board consisted of four sensor signals, namely the three cutting force components (*F_x_*, *F_y_*, *F_z_*) and the Root Mean Square of the acoustic emission (*AE_RMS_*) signal. As regards the vibration acceleration signals, the wireless digital sensor employed in this research work was provided with embedded low-pass filters designed to remove the high-frequency noise. The digital sensor performed signal sampling with a fixed rate of 3.24 kS/s and directly sent to the PC via wireless connection the sensorial data corresponding to the three vibration acceleration components (*A_x_*, *A_y_*, *A_z_*).

### 2.2. Tool Wear Measurement

**Figure 2:**
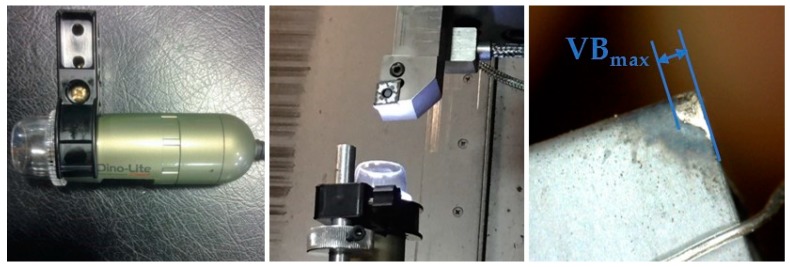
Portable digital microscope and measurement of maximum flank wear land, *VB_max_*. Cutting parameters: *v* = 60 m/min, *f* = 0.2 mm/rev, *d* = 1 mm (pass no. 4).

In order to construct the tool wear curve for each turning test, the testing procedure was performed according to the requirements of the ISO 3685:1993 international standard on tool-life testing with single-point turning tools, by acquiring at least 5 tool wear values for each tool wear curve [21]. After each turning pass, a magnified picture of the cutting insert was captured in-place by means of a portable digital microscope (DINO-LITE Premier) without removing the cutting tool from the tool holder and the maximum flank wear land, *VB_max_*, was measured, as shown in Figure 2. A threshold was set to the maximum acceptable flank wear value, *VB_max_* = 0.6 mm and it was used as a criterion for identifying the end of tool life.

**Figure 3:**
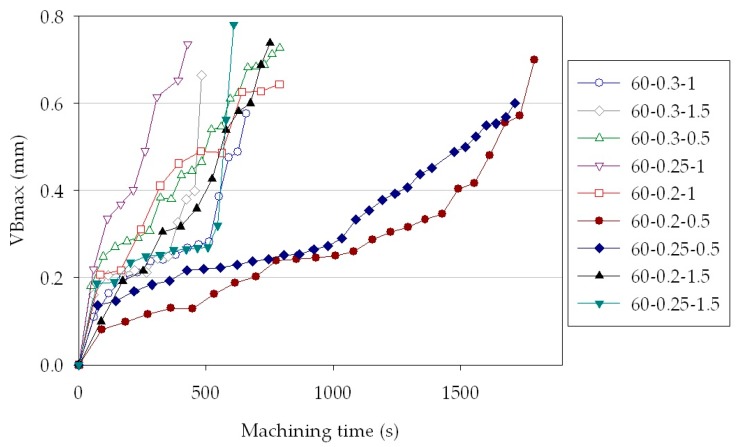
Measured maximum tool flank wear values vs. machining time for all the cutting conditions.

The influence of depth of cut and feed rate variations on tool wear development can be observed in Figure 3. A remarkable increase of tool wear rate, almost three times faster, was displayed when augmenting the depth of cut from *d*_1_ = 0.5 mm to *d*_2_ = 1 mm and *d*_3_ = 1.5 mm as well as when increasing the feed rate from *f*_2_ = 0.25 mm/rev to *f*_3_ = 0.3 mm/rev.

## 3. Sensor Signal Pre-Processing

Sensor signal pre-processing, including signal conditioning and segmentation, was carried out on the sensor signals acquired by the multiple sensor system during the experimental turning tests.

The force and the acoustic emission signals were conditioned to take into account the gain settings of the sensor signal amplifiers, while the vibration acceleration signals were resampled to 10 kS/s to match the sampling rate of the DAQ signals. Moreover, each vibration acceleration component signal (*A_x_*, *A_y_*, *A_z_*) was shifted to remove the offset which was due to its relative orientation with respect to the gravity acceleration (the sum of the *x*, *y* and *z* offset values is 9.81 m/s^2^). 

**Figure 4:**
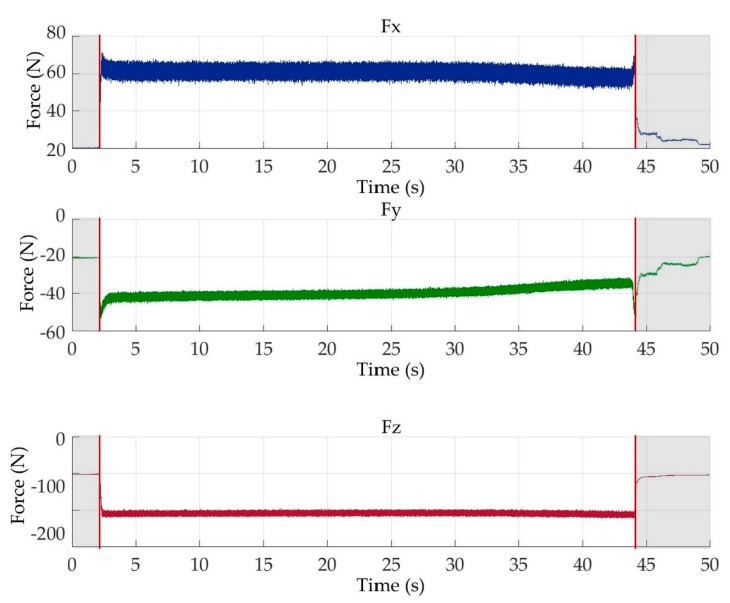
Example of cutting force signals, *F_x_*, *F_y_*, *F_z_* and segmented signal portions.

**Figure 5:**
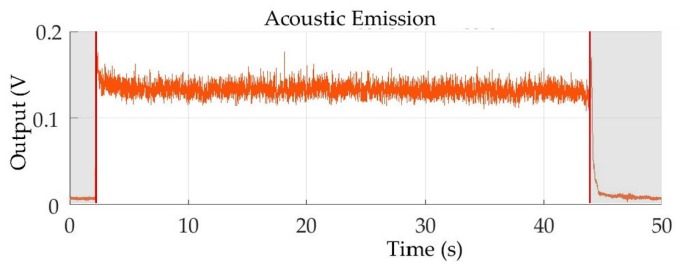
Example of acoustic emission RMS signal, *AE_RMS_* and segmented signal portions.

Afterwards, segmentation was carried out on all the acquired signals in order to remove the signal portions not corresponding to the actual machining process, such as the signal head and tail, related to the phases of tool approaching and moving away from the workpiece, respectively [5,6]. Automated segmentation was performed by calculating the signal moving average with a fixed subset size of 50 samples to reduce high frequency oscillations and employing a threshold to identify the actual machining signal portion. Considering only the part of the signal over the threshold, the slope between consecutive samples was calculated: the first sample with negative slope and the last sample with positive slope were identified as the start and the end of the segmented signal. This segmentation was performed on the *F_x_* signal and extended to the synchronized *F_y_*, *F_z_* and *AE_RMS_* signals (Figure 4 and Figure 5).

**Figure 6:**
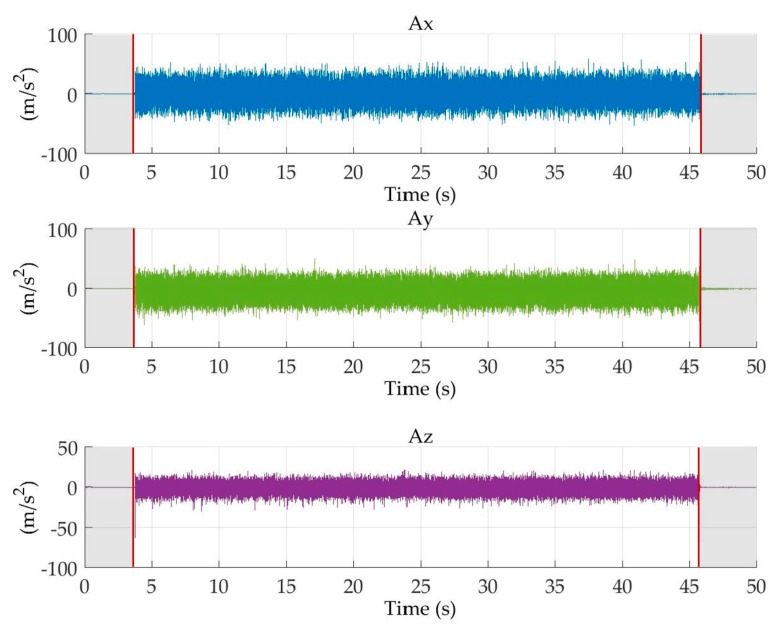
Example of vibration acceleration signals, *A_x_*, *A_y_*, *A_z_* and segmented signal portions.

A different segmentation procedure was applied to the acceleration signals acquired by the digital vibration sensor. The developed procedure identified the difference between transient and actual machining signal portions by calculating the square of the signal. The first and the last value over the threshold (set at 20 m/s^2^ based on the analysis of the signals) were selected as the start and the end of the signal segment related to actual machining (Figure 6).

## 4. Sensor Signal Features Extraction

With the aim to obtain sensor signal features functional for the tool condition monitoring scope, a statistical procedure for feature extraction in the time domain was applied [21]. 

From each of the seven acquired sensor signals, four statistical signal features describing the signal in the time domain, namely arithmetic mean, variance, skewness and kurtosis, were extracted, generating a total of 28 sensor signal features for each turning pass, as shown in Table 2.

The statistical features, mean, variance, skewness and kurtosis, represent the first, second, third and fourth moment of the sensor signals, respectively and they were selected as they allow to describe the shape of the set of acquired sensorial data points. The data set comprising the extracted features for each turning pass should be fed to a machine learning system based on artificial neural networks to provide a diagnosis on tool wear conditions. However, the large dimensionality of this sensorial features data set could increase the complexity of the machine learning algorithm. As a matter of fact, the complexity of any classifier or regressor depends on the number of inputs, which determines both the time and space complexity and the required number of training samples for the learning algorithm [22].

## 5. Feature Dimensionality Reduction

Reducing dimensionality is an important pre-processing step for machine learning due to several reasons. First, in most learning algorithms, the complexity depends on the number of input dimensions as well as on the size of the data sample, so that, to reduce memory and computation, it is necessary to reduce the dimensionality of the problem. Decreasing the input dimensions also decreases the complexity of the inference algorithm during testing. Moreover, when an input is proved to be unnecessary, the cost for its extraction can be saved. Simpler models are also more robust on small datasets and have less variance, that is, they vary less depending on noise, outliers, etc. When data can be explained with fewer features, it is easier to get an idea about the process that underlies the data and this allows knowledge extraction. Finally, when data can be represented in a few dimensions without loss of information, they can be plotted and analysed visually for structure and outliers [23,24]. Therefore, in machine learning, it is essential to perform feature set dimensionality reduction.

In general, a reduction in the dimensionality of the input space may cause a loss of some of the information which discriminates between different classes or determines the target values. The goal in dimensionality reduction is therefore to preserve as much of the relevant information as possible via proper methods of feature selection, choosing a subset of important features cutting off the others and feature extraction, generating fewer, new features from the original ones [23,24]. 

In feature selection, the objective is to find *k* of the original *d* dimensions (*k* < *d*) that provide the most information and discard the other (*d* − *k*) dimensions. In feature extraction, the aim is to find a new set of *k* dimensions that are combinations of the original *d* dimensions. These methods may be supervised or unsupervised depending on whether or not they use the output information.

In this work, feature set dimensionality reduction was performed by using first supervised feature selection to cut off irrelevant features and then advanced unsupervised feature extraction based on linear projection via Principal Components Analysis (PCA) to combine the relevant features into a lower number of new features.

### 5.1. Feature Selection Based on Pearson’s Correlation

A filter method for feature selection based on the Pearson’s correlation coefficient was employed to evaluate the correlation between extracted features and tool wear conditions. The Pearson’s correlation coefficient, *r*, between a feature *x* and tool wear value *y* was calculated as follows:

(1) $$r=\frac{\sum_{i=1}^{n}\left(x_{i}-\bar{x}\right)\left(y_{i}-\bar{y}\right)}{\sqrt{\sum_{i=1}^{n}{\left(x_{i}-\bar{x}\right)}^{2}}\sqrt{\sum_{i=1}^{n}{\left(y_{i}-\bar{y}\right)}^{2}}}\;$$

The value of *r* allows to classify the level of correlation according to three classes: weak correlation (0 < *r* < 0.3), moderate correlation (0.3 < *r* < 0.7) and strong correlation (0.7 < *r* < 1). 

Feature selection was performed by choosing only the statistical features having a strong correlation with tool wear on the basis of the *r* value (*r* > 0.7): *F_x_* average (*F_x_av_*), *F_y_* average (*F_y_av_*), *F_z_* average (*F_z_av_*), AE_RMS_ average (*AE_RMS_av_*), *F_x_* skewness (*F_x_sk_*), *F_z_* skewness (*F_z_sk_*), *F_x_* kurtosis (*F_x_kurt_*), *F_z_* kurtosis (*F_z_kurt_*). In this way, the number of features was initially cut from 28 to 8 features by removing those which did not display a strong correlation with the output.

### 5.2. Features Extraction via Principal Component Analysis 

To further cut the number of features without experiencing actual loss of information, a feature extraction method based on linear projection via Principal Components Analysis (PCA), which is one of the most widely used feature extraction methods for dimensionality reduction, was employed [23,24,25].

PCA is an unsupervised linear projection method, allowing to perform a mapping from the input vectors ***x*** in the original *d*-dimensional space to new vectors ***z*** in the *k*-dimensional space (with *k* < *d*), with minimum loss of information. PCA identifies new variables along new directions, i.e., the principal components, which are linear combinations of the original variables. 

The projection of ***x*** on the direction of ***w*** is:

(2) $$z=\;w^{T}x\;$$

PCA is an unsupervised method as it does not use the output information; as a matter of fact, the criterion to be maximized is the variance. If data are standardized (mean = 0), the principal components are calculated as the normalized eigenvectors of the covariance matrix of the original variables and they are ordered according to how much of the variation present in the data they contain. Each component can then be interpreted as the direction, uncorrelated to previous components, which maximizes the variance of the samples when they are projected onto the component itself.

Therefore, the principal component, *w*_1_, is the eigenvector of the covariance matrix of the input sample with the largest eigenvalue, *λ*_1_, i.e., the direction along which the samples show the largest variation. The second principal component, *w*_2_, is the direction uncorrelated to the first component with the largest eigenvalue, *λ*_2_ and so on until a total of *d* principal components, equal to the original number of variables, have been calculated. At the end, the sum of the variances of all of the principal components will equal the sum of the variances of all of the variables, that is, all of the original information has been explained.

The positions of each observation in this new coordinate system of principal components are called scores and are calculated as linear combinations of the original variables and the relative weights.

When *λ_i_* are sorted in descending order, the proportion of variance explained by the *k* principal components is:

(3) $$\frac{\lambda_{1}+\lambda_{2}+\ldots +\lambda_{k}}{\lambda_{1}+\lambda_{2}+\ldots +\lambda_{k}+\ldots +\lambda_{d}}\;$$

Given a set of data vectors in a *d*-dimensional space, if the first *k* eigenvalues have significantly larger values than the remaining *d − k* eigenvalues, it means that the data can be represented to a relatively high accuracy by projection onto the first *k* eigenvectors. Therefore, the effective dimensionality of the data is less than the apparent dimensionality *d,* as a result of correlations within the data. Accordingly, only the eigenvectors corresponding to the *k* largest eigenvalues are retained and the input vectors ***x*** are projected onto the eigenvectors to give the components of the transformed vectors ***z*** in the *k*-dimensional space. If the dimensions are highly correlated, there will be a small number of eigenvectors with large eigenvalues and *k* will be much smaller than *d* and a large reduction in dimensionality may be attained. If the dimensions are not correlated, *k* will be as large as *d* and there is no gain through PCA.

**Figure 7:**
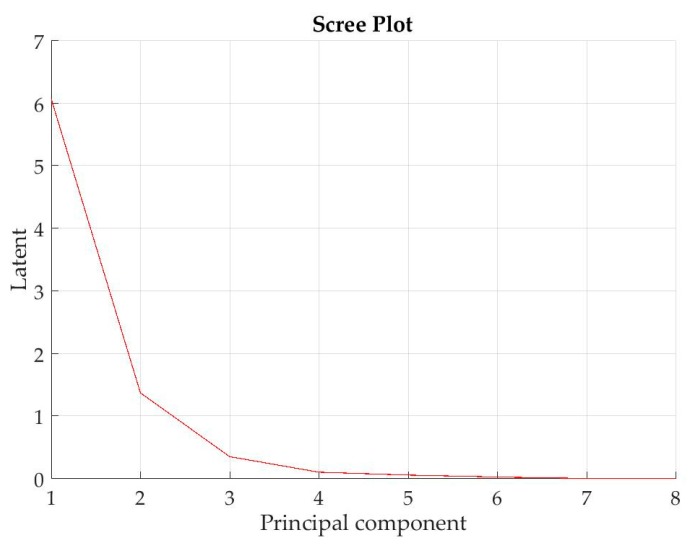
Scree plot reporting the variance explained as a function of the principal components for test 8 (*v* = 60 m/min, *f* = 0.3 mm/rev, *d* = 1 mm).

In this work, PCA was applied via Singular Value Decomposition (SVD), which is a computationally efficient method for finding principal components. Through linear projection from the *d* = 8 original statistical features, *d* = 8 principal components were generated (herewith called *PC*1, …, *PC*8). To decide the suitable size of *k* (*k < d*) allowing for feature reduction without loss of important information, the scree graph method was employed. The scree graph is the plot of variance explained (i.e., the eigenvalues of the covariance matrix of ***x***, returned as a column vector) as a function of the number of eigenvectors (i.e., the principal components) and is used to decide on the size of *k* via visual analysis. When the plot takes a bend displaying an “elbow,” it indicates that adding another eigenvector does not considerably increase the variance explained. Figure 7 shows the scree plot reporting the variance explained as a function of the principal components for test U1-L3 (*v* = 60 m/min, *f* = 0.3 mm/rev, *d* = 1 mm). It can be observed that the elbow occurs between the 2nd and 3rd principal components, suggesting that a number of *k* = 2 components is sufficient to describe the variance of the data. The same behaviour was observed in the scree plots of the other turning tests, accordingly, for each test, a number of 2 principal components were selected to be used for machine learning. Specifically, the scores, i.e., the representations of the original data in the principal component space (where rows correspond to observations and columns correspond to components), corresponding to the first 2 principal components were used as input for machine learning. The principal components scores represent sensor fusion features, as they are linear combinations of the original features extracted from the multiple sensor signals of different nature (force and acoustic emission, respectively).

The analysis of the principal components coefficients allows to evaluate the contribution of each original statistical feature in the linear combination characterizing every principal component. In particular, over all the tests, it was observed that the first principal component was characterized by higher coefficients for the *F_x_* average, *F_y_* average and *AE_RMS_* average, while the second component was generally characterised by higher coefficient relative to *F_z_* skewness (*F_z_sk_*) and *F_z_* kurtosis (*F_z_kurt_*).

In this way, a significant dimensionality reduction was achieved, decreasing the number of required features from the initial 28 statistical features to 8 features via statistical correlation and finally to 2 features via PCA, cutting off irrelevant features and combining the significant ones without losing important information. In practice, each of the 9 experimental tests in Table 1 was initially represented by a set of *p* data vectors (where *p* is the number of turning passes) in a *d*-dimensional space (*d* = 28 features for each turning pass). After the implementation of the PCA method, the data set for each experimental test was drastically reduced to a set of *p* vectors in a smaller *k*-dimensional space (*k* = 2 principal components for each turning pass *p_i_*).

**Figure 8:**
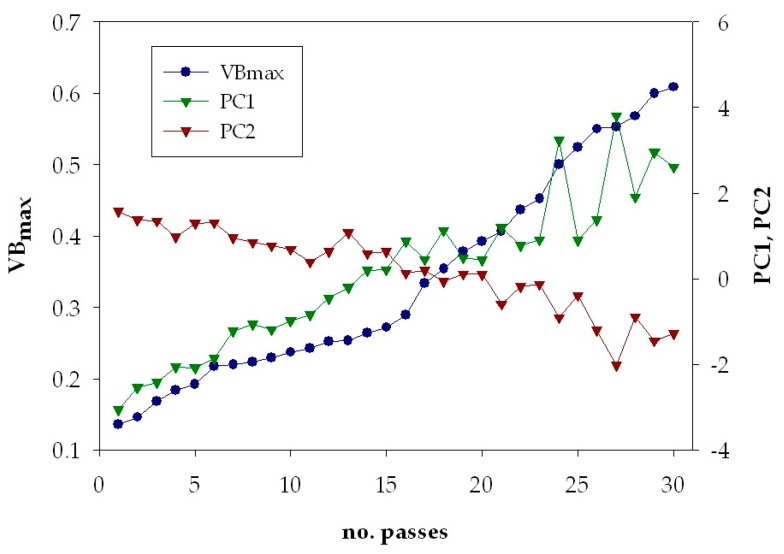
Scores of the first two principal components, *PC*1 and *PC*2 and measured tool wear values, *VB_max_*, vs. number of turning passes for test 4 (*v* = 60 m/min, *f* = 0.25 mm/rev, *d* = 0.5 mm).

Graphical analysis of the first two principal components scores plotted together with the corresponding tool wear value, *VB_max_*, shows that their behaviour with increasing number of turning passes seems to reflect the tool wear development. Figure 8 illustrates the *PC*1 and *PC*2 principal components scores and the tool wear values for the cutting test carried out at *v* = 60 m/min, *f* = 0.25 mm/rev and *d* = 0.5 mm. It can be observed that *PC*1 has an increasing trend, while *PC*2 has a decreasing trend and both seem to follow the tool wear development.

## 6. Machine Learning for Tool Wear Diagnosis Based on Artificial Neural Network

Machine learning based on artificial intelligence methods allows for data-driven formulation of complex models able to make predictions or decisions from sample inputs by revealing the structural patterns embedded in data [26]. With the aim to estimate the tool wear state through cognitive pattern recognition based on the input PCA features extracted from the sensor signals, a machine learning model based on artificial neural networks (ANN) was developed [24,27]. 

The two selected PCA features, *PC*1 and *PC*2, were employed to construct sensor fusion feature pattern vectors (SFPVs) to be fed to the ANN for pattern recognition [5]. 

For each turning pass *i*, a 3-feature SFPV was built by combining the two selected PCA features with the corresponding time, i.e., the machining time cumulated at the end of that turning pass.

(4) $$SFPV_{i}=\left[PC1_{i},PC2_{i},t_{i}\right]\;i=1,\;\ldots ,\;p$$

where *p* is the number of turning passes and *t_i_* the time cumulated at the end of the turning pass.

In this way, for each turning test of Table 1, a learning set consisting of a number of *p* SFPVs, equal to the number of turning passes, was set up.

For each turning test, ANNs with different architectures were set up in order to reconstruct the complete tool wear curve using the SFPV training set.

Supervised machine learning was implemented by associating each 3-feature SFPV to the corresponding maximum flank wear land, *VB_max_*.

The artificial neural networks employed for machine learning in this research work were cascade-forward neural networks. The latter are multilayer perceptrons (MLP) that can approximate the nonlinear functions of the input and they are characterized by connections going from the input and every previous layer to the following layers.

Three-layer cascade-forward backpropagation ANNs were built with a number of input layer nodes equal to 3, that is the number of input features of each SFPV and different numbers of hidden layer nodes, equal to 3, 6 and 9 nodes, i.e., 1×, 2× and 3× the number of input layer nodes, with the aim to find the best ANN configuration providing the highest performance rate. As a matter of fact, when the network is too large and has too many free parameters, generalization may not be well performed, therefore, to find the optimal network size, the most common approach is to try different architectures with different hidden nodes [23]. The output layer had a number of nodes equal to 1, corresponding to the maximum tool wear land, *VB_max_*. The Levenberg-Marquardt optimization algorithm was chosen as ANN training function, while the tan-sigmoid was selected as transfer function. The number of epochs was set equal to 1000 and the minimum performance gradient was set to 1 × 10^−7^.

ANN cross-validation was performed through the leave-k-out method with *k* = 1 [27]. According to the leave-k-out method, at each step, *k* = 1 SFPV was removed in turn from the original set of *p* SFPVs and used for ANN testing while the remaining *p*−*k* SFPVs were used for training. This procedure was repeated for all the *p* SFPVs and the overall pattern recognition performance was eventually estimated by aggregating the *p* recognition rates obtained.

## 7. Results

The tool wear diagnosis performance achieved by the different ANN architectures was estimated in terms of mean squared error, MSE, between the *VB_max_* values predicted by the ANN and the measured *VB_max_* values, calculated as follows:

(5) $$MSE=\frac{1}{n}\overset{n}{\underset{i=1}{\sum }}{\left(VB_{max}^{pred}-VB_{max}^{meas}\right)}^{2}\;$$

**Figure 9:**
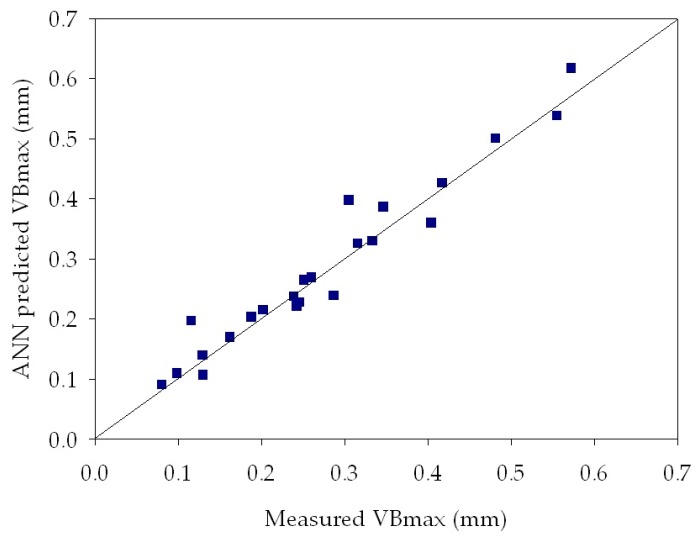
Regression plot between ANN predicted and measured *VB_max_* for turning test 1 at *v* = 60 m/min, *f* = 0.20 mm/rev, *d* = 0.5 mm. ANN configuration: 3-6-1. MSE = **2.48** **× 10^−3^**.

**Figure 10:**
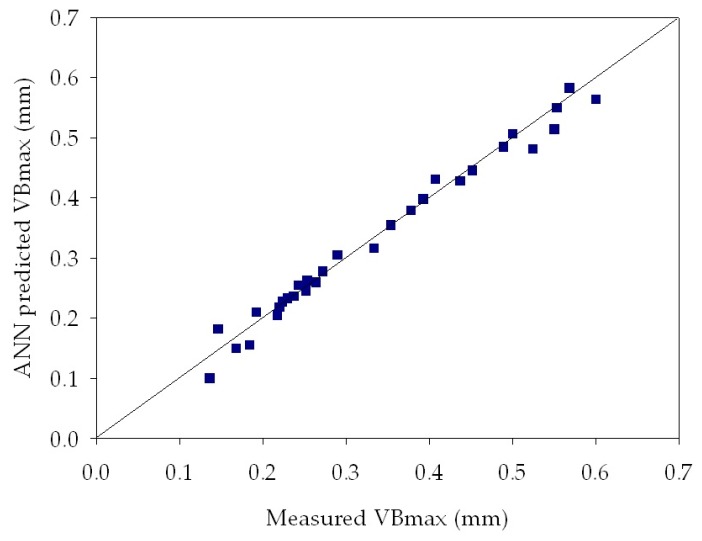
Regression plot between ANN predicted and measured *VB_max_* for turning test 4 at *v* = 60 m/min, *f* = 0.25 mm/rev, *d* = 0.5 mm. ANN configuration: 3-3-1. MSE = **8.12** **× 10^−4^**.

**Figure 11:**
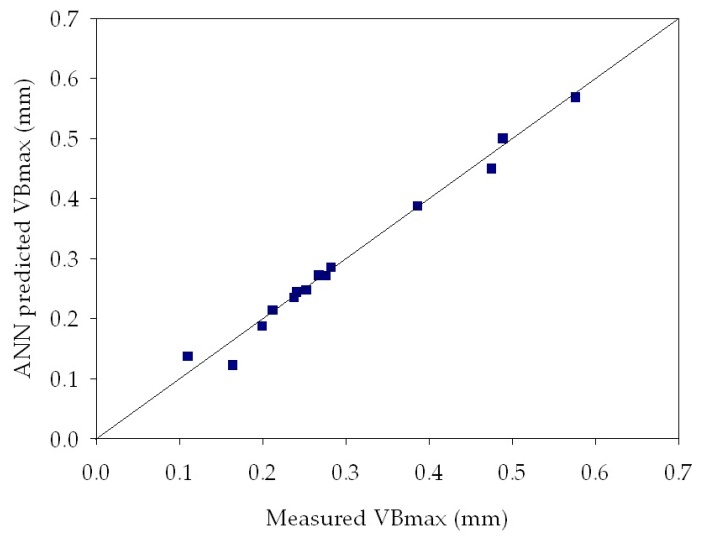
Regression plot between ANN predicted and measured *VB_max_* for turning test 8 at *v* = 60 m/min, *f* = 0.30 mm/rev, *d* = 1.0 mm. ANN configuration: 3-6-1. MSE = **2.54** **× 10^−4^**.

Table 3 reports MSE values obtained by the ANNs for tool wear estimation of all the experimental turning tests. It can be observed that very low MSE values, between 2.54 × 10^−4^ and 5.17 × 10^−2^, were achieved for all the experimental tests. These low values indicate that the ANN output values are very close to the measured maximum flank wear values, therefore the ANN provided an accurate tool wear diagnosis. Table 3 also shows the influence of the varying number of hidden layer nodes: in most cases, the best performance was obtained with a lower number of hidden nodes, namely with the 3-3-1 and the 3-6-1 ANN configurations. In Figure 9, Figure 10 and Figure 11, the ANN predicted *VB_max_* values are reported vs. the measured *VB_max_* values for some of the performed experimental tests. In each graph, the diagonal line indicates the ideal condition in which the measured *VB_max_* and the predicted *VB_max_* coincide: the dispersion of values around this line allows to graphically evaluate the ANN tool wear prediction performance, which shows to be very accurate.

## 8. Conclusions

With the aim to monitor the tool conditions during dry turning of Ti-6Al-4V alloy, a machine learning procedure based on the acquisition and processing of cutting force, acoustic emission and vibration sensor signals during turning was implemented. 

The acquired sensor signals were processed to extract a number of sensorial features (*d* = 28 statistical features) in order to feed machine learning paradigms based on artificial neural networks. To reduce the large dimensionality of the sensorial features, an advanced feature extraction methodology based on Principal Component Analysis was proposed.

PCA allowed to identify a smaller number of features (*k* = 2 features), the principal component scores, obtained through linear projection of the original *d* features into a new space with reduced dimensionality *k* = 2, which proved to be sufficient to describe the variance of the data. The extracted principal components scores represented sensor fusion features, being linear combinations of the original features extracted from the multiple sensor signals of different nature.

By feeding machine learning algorithms based on artificial neural networks with the PCA features, an accurate diagnosis of tool wear (maximum flank wear, *VB_max_*) was achieved, with ANN predicted values very close to the measured tool wear values and mean squared error <5.17 × 10^−2^.

The accurate tool wear diagnosis obtained through the machine learning procedure presented in this work could be effectively implemented on-line to monitor the tool conditions during the turning process and support more efficient condition-based tool replacement strategies. The dimensionality reduction performed on the sensorial features data set improved the efficiency of the machine learning procedure, by lowering the complexity of the models and drastically reducing the number of data to be stored, which is a major goal in today’s Industry 4.0 framework.

The developed procedure could be extended to other machining processes under different cutting conditions and different workpiece materials, provided the availability of training sets for tool wear diagnosis obtained via further experimental campaigns.

## Figures and Tables

**Table 1:** Cutting conditions and number of passes for each experimental turning test.

Test ID	Cutting Speed (m/min)	Feed Rate (mm/rev)	Depth of Cut (mm)	No. of Passes
Test 1	60	0.20	0.5	24
Test 2	60	0.20	1.0	10
Test 3	60	0.20	1.5	12
Test 4	60	0.25	0.5	30
Test 5	60	0.25	1.0	8
Test 6	60	0.25	1.5	12
Test 7	60	0.30	0.5	20
Test 8	60	0.30	1.0	14
Test 9	60	0.30	1.5	11

**Table 2:** Extracted statistical sensor signal features for each turning pass.

Sensor Signal	*F_x_*	*F_y_*	*F_z_*	*AE_RMS_*	*A_x_*	*A_y_*	*A_z_*
Extracted features per pass	*F_x_mean_*, *F_x_var_*, *F_x_skew_*, *F_x_kurt_*	*F_y_mean_*, *F_y_var_*, *F_y_skew_*, *F_y_kurt_*	*F_z_mean_*, *F_z_var_*, *F_z_skew_*, *F_z_kurt_*	*AE_RMS_mean_*, *AE_RMS _var_*, *AE_RMS_skew_*, *AE_RMS_kurt_*	*A_x_mean_*, *A_x_var_*, *A_x_skew_*, *A_x_kurt_*	*A_y_mean_*, *A_y_var_*, *A_y_skew_*, *A_y_kurt_*	*A_z_mean_*, *A_z_var_*, *A_z_skew_*, *A_z_kurt_*

**Table 3:** Overall Mean Square Error (MSE) obtained by ANN tool wear estimation for all the turning tests.

		MSE	
	3 Hidden Nodes	6 Hidden Nodes	9 Hidden Nodes
Test 1	5.31 × 10^−3^	**2.48 × 10^−3^**	2.51 × 10^−2^
Test 2	5.22 × 10^−3^	8.66 × 10^−3^	**2.94 × 10^−3^**
Test 3	**2.93 × 10^−3^**	5.48 × 10^−3^	1.31 × 10^−2^
Test 4	**8.12 × 10^−4^**	1.95 × 10^−3^	1.87 × 10^−3^
Test 5	2.23 × 10^−3^	**1.63 × 10^−3^**	6.56 × 10^−3^
Test 6	**2.12 × 10^−2^**	3.69 × 10^−2^	5.17 × 10^−2^
Test 7	8.67 × 10^−3^	**4.19 × 10^−4^**	3.71 × 10^−2^
Test 8	**2.54 × 10^−4^**	1.22 × 10^−3^	3.20 × 10^−3^
Test 9	3.48 × 10^−3^	**2.16 × 10^−3^**	3.02 × 10^−3^
